# BRAF Mutations in Melanoma: Biological Aspects, Therapeutic Implications, and Circulating Biomarkers

**DOI:** 10.3390/cancers15164026

**Published:** 2023-08-08

**Authors:** Giorgia Castellani, Mariachiara Buccarelli, Maria Beatrice Arasi, Stefania Rossi, Maria Elena Pisanu, Maria Bellenghi, Carla Lintas, Claudio Tabolacci

**Affiliations:** 1 Department of Oncology and Molecular Medicine, Istituto Superiore di Sanità, 00161 Rome, Italy; giorgia.castellani@iss.it (G.C.); mariachiara.buccarelli@iss.it (M.B.); mariabeatrice.arasi@iss.it (M.B.A.); stefania.rossi@iss.it (S.R.); 2 High Resolution NMR Unit, Core Facilities, Istituto Superiore di Sanità, 00161 Rome, Italy; mariaelena.pisanu@iss.it; 3 Center for Gender-Specific Medicine, Istituto Superiore di Sanità, 00161 Rome, Italy; maria.bellenghi@iss.it; 4 Research Unit of Medical Genetics, Department of Medicine, Università Campus Bio-Medico di Roma, 00128 Rome, Italy; clintas@unicampus.it; 5 Operative Research Unit of Medical Genetics, Fondazione Policlinico Universitario Campus Bio-Medico, 00128 Rome, Italy

**Keywords:** melanoma, BRAF mutations, BRAF V600, targeted therapy resistance, biomarkers

## Abstract

**Simple Summary:**

Cutaneous melanoma represents the most aggressive form of skin cancer and its occurrence, development, and progression are based on the accumulation of several genetic alterations. BRAF mutations are frequently found in melanoma and targeted therapies against these specific genetic modifications have significantly changed the management of melanoma patients. However, these treatments are often associated with the induction of resistance. Therefore, this review aims to summarize recent findings on the impact of BRAF mutations on different aspects of melanomagenesis, including inflammation. Furthermore, we provide an overview of the main mechanisms of resistance to BRAF inhibitors and circulating tumour biomarkers.

**Abstract:**

Melanoma is an aggressive form of skin cancer resulting from the malignant transformation of melanocytes. Recent therapeutic approaches, including targeted therapy and immunotherapy, have improved the prognosis and outcome of melanoma patients. BRAF is one of the most frequently mutated oncogenes recognised in melanoma. The most frequent oncogenic BRAF mutations consist of a single point mutation at codon 600 (mostly V600E) that leads to constitutive activation of the BRAF/MEK/ERK (MAPK) signalling pathway. Therefore, mutated BRAF has become a useful target for molecular therapy and the use of BRAF kinase inhibitors has shown promising results. However, several resistance mechanisms invariably develop leading to therapeutic failure. The aim of this manuscript is to review the role of BRAF mutational status in the pathogenesis of melanoma and its impact on differentiation and inflammation. Moreover, this review focuses on the mechanisms responsible for resistance to targeted therapies in BRAF-mutated melanoma and provides an overview of circulating biomarkers including circulating tumour cells, circulating tumour DNA, and non-coding RNAs.

## 1. Introduction

Cutaneous melanoma represents the most lethal form of skin cancer resulting from the malignant transformation of melanocytes. Melanocytes are responsible for the production of a pigment, melanin, which can be transferred to neighbouring keratinocytes with accumulation in the supranuclear region of these cells protecting them from ultraviolet (UV)-induced DNA damage [1]. There are two main types of melanin, differing in color and photo-protection: (i) brown-black melanin called eumelanin that is UV absorbent; (ii) red melanin named pheomelanin that is photo-unstable [2,3]. The synthesis of both pigments begins with the hydroxylation of tyrosine to dihydroxyphenylalanine and subsequent oxidation to dopaquinone by tyrosinase (TYR) activity [2]. Eumelanin can be deposited onto melanosomal fibrils consisting mainly of PMEL17/gp100, a specific marker of melanocytes, and suggested as a target for anti-melanoma therapy [4].

Melanoma, a relatively rare tumour accounting for less than 2% of global cancer diagnoses, is responsible for 80% of skin cancer deaths with a growing incidence over past decades especially in the Caucasian population [5]. The etiopathogenesis of melanoma is closely linked to genetic, epigenetic, and environmental risk factors. The prolonged exposure to the sun (also due to specific geographical locations) and the use of tanning beds or sun lamps are major environmental melanoma causes since they represent a principal source of UVB radiation, which produces skin damage in a dose-dependent manner [6,7]. In particular, UV radiation promotes melanomagenesis by causing direct DNA damage with resulting pro-carcinogenic gene mutations, triggering inflammation and oxidative stress, and stimulating immunosuppressive molecules [8,9]. Therefore, people with pale skin, freckles, and light or red hair have a higher risk of developing melanoma than people with dark hair and eyes [10]. Interestingly, several lines of evidence point to a close relationship between oxidative stress and epigenetic mechanisms (inherited changes in gene expression resulting from chemical alterations of DNA without nucleotide variations) that may be involved in cancer development [11]. Alterations in the production of microRNAs (miRNAs) and other non-coding RNA (ncRNA) species, histone modifications, and aberrant DNA methylation are associated with several phases of melanoma initiation and progression including drug resistance and anti-tumour immunity [12,13]. However, in addition to environmental and phenotypic predisposition, genomic instability is the major risk factor for melanoma. The widespread use of sequencing strategies such as next-generation sequencing (NGS) technologies has accelerated the identification of somatic mutations of melanoma [14]. Several key genetic alterations in known (e.g., *BRAF*, *NRAS*, *MITF*, *KIT*, *TP53*, *CDKN2A*, and *PTEN*) and recently identified (e.g., *NF1*, *RAC1*, *IDH1*, and *ARID2*) genes have been described as crucial factors in melanoma development [14,15]. Among these mutations, *BRAF* and *NRAS* are two of the most common, but also mutually exclusively mutated, oncogenes recognised in melanoma. Mutations in BRAF promote melanomagenesis through the constitutive activation of the mitogen-activated protein kinase (MAPK) pathway leading to increased cell proliferation [16]. In fact, the MAPK (RAS/RAF/MEK/ERK) pathway is activated by various stimuli (growth factors, cytokines, and hormones) that bind the membrane receptor with tyrosine kinase activity (receptor tyrosine kinase, RTK), leading to final activation of ERK. Thus, ERK migrates into the nucleus where it can recognise and phosphorylate several transcription factors and protein kinases [17]. In this review, we will discuss various biological aspects of melanoma related to BRAF mutation, including circulating and tissue biomarkers. We will also describe the mechanisms responsible for resistance to targeted therapies in BRAF-mutated melanoma.

## 2. BRAF Mutations as Key Players of Genetic Instability in Melanoma

**Figure 1:**
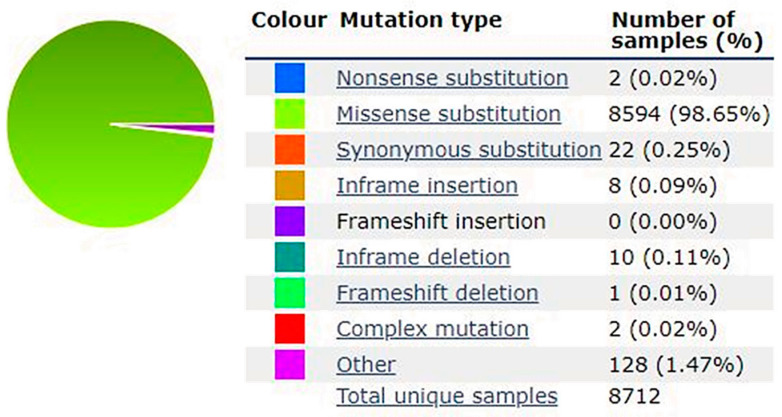
Types of *BRAF* variants detected in melanoma as reported in the COSMIC database (https://cancer.sanger.ac.uk/cosmic/ (accessed on 22 May 2023)).

Pathogenetic variants of the oncogene BRAF are dominant and activate mutations that affect the MAPK pathway. As stated above, the constitutively activated BRAF promotes tumour cell proliferation and survival but also cell invasion, metastasis, and evasion of the immune response [18]. In addition to melanoma, other cancer types such as non-Hodgkin’s lymphoma, colorectal carcinoma, thyroid carcinoma, non-small cell lung carcinoma, hairy cell leukemia, and lung adenocarcinoma harbor pathogenetic variants in this gene [19,20]. Germinal variants in this gene are also associated with cardiofaciocutaneus Noonan and Leopard syndromes, two developmental disorders [21]. In melanomas, the somatic BRAF mutation rate is the highest among cancer types and pathogenetic variants in the *BRAF* (OMIM*164757) gene occur in about 50% of melanoma-affected patients. BRAF mutations are more frequent in young patients who are only occasionally exposed to the sun than in chronically sun-exposed individuals. BRAF-mutated melanomas display unique features: they tend to be more aggressive than BRAF wild-type (WT) melanomas, are more likely to metastasize to the brain, and are associated with shorter survival in patients with stage IV tumours [17]. In addition to *BRAF*, other genes have been found mutated in sporadic melanomas including *NRAS*, *TERT*, *NF1*, and *KIT* [22]. In hereditary forms of melanoma, which represent about 10% of melanomas, no BRAF mutations have ever been described to date. Melanoma *BRAF* mutations are nearly all missense variants (98.65%) as reported in the COSMIC (Catalogue of Somatic Mutation in Cancer) database (Figure 1).

*BRAF* pathogenetic variants mostly involve codon 600 [16,23] which is located within the kinase domain (Table 1).

The amino acid change at codon 600 results in an increase in the kinase activity which is a 480-fold increase for the specific most common V600E change [24]. This variant accounts for 70–88% of BRAF-positive melanomas and it is a missense variant consisting of a substitution of valine with glutamic acid. The second most common variant is p.V600K, consisting of the substitution of valine with lysine, and occurring at a frequency of about 10–20%. V600K-positive melanomas appear more aggressive with respect to V600E-positive melanomas and have a higher mutational burden. The variants p.V600R, consisting in the substitution of valine with arginine, and p.V600D consisting in the substitution of valine with aspartic acid, are two other very rarely detected *BRAF* mutations accounting for about less than 5% each. The variants p.V600E2, p.V600M, and p.V600G have also been detected in less than 1% of cases. In general, BRAF V600 variants respond to specific inhibitors that improve the overall survival (OS) in patients with metastatic cutaneous melanoma. Non-V600 *BRAF* mutations have occasionally been reported in melanomas (Table 1) and they account for approximately 11% of melanomas [25]. Mutations involving L597 and K601 codons are located in the activation segment domain whereas the G469 codon falls within the glycine-rich region of the protein. Like V600 variants, these mutations in these codons increase the BRAF kinase activity but in a different way and are insensitive to BRAF inhibitors (BRAFi). Finally, variants involving the D594 and the G596 codons decrease or impair the BRAF kinase activity [25]. Another type of genetic rearrangement commonly observed in melanomas is *BRAF* gene fusions, which have been detected in 3-6% of patients and are more frequent in young women with certain histopathological subtypes. *BRAF* gene fusions are predominately intra-chromosomal translocations that result in the loss of the auto-inhibitory domain but retention of the kinase domain [26], leading to constitutive activation of the protein.

At the morphological and histological level, melanoma can be mainly distinguished into major subtypes with different prevalences in the general population, including superficial spreading melanoma (most common in the Caucasian population), nodular melanoma, lentigo maligna melanoma, and acral lentiginous melanoma (most common in dark skin and Asian populations) [27]. Other rare subtypes are mucosal, desmoplastic, and uveal melanomas. It is interesting to note that different melanoma subtypes are characterized by different frequencies of *BRAF* and *NRAS* mutations [28,29]. In fact, a low mutation rate of *BRAF* was observed in acral melanomas than in non-acral melanomas [30].

Mutational burden has been correlated with gender in some but not all reports [31,32]. Gupta et al. reported in a cohort of 266 metastatic melanoma patients that missense variants were more common in males than females (298 missense variants in males compared to 211 missense variants in females) [33]. These data were also confirmed in a separate cohort of 95 melanoma patients in which missense variants were 293 and 259 for male and female patients, respectively. As far as specific *BRAF* mutations concern, Van der Kooij and colleagues [34] analyzed 3985 patients with advanced melanoma: BRAF V600E mutation was more frequent in women (46% females versus 36% males) in all age groups analyzed; on the other hand, BRAF V600K mutation was prevalent in male patients (8% males versus 4% females) regardless of age. Lokhandwala et al. [35] did not find a statistically significant association between specific BRAF mutations and gender, though they confirmed a higher incidence of *BRAF* V600K mutation in male patients. They also reported enrichment of mutants not involving codon 600 and mutations not affecting BRAF kinase activity in males. In another study, based on 319 Sardinian melanoma patients, no significant differences in BRAF mutation distribution between genders could be detected [36].

## 3. Biological Aspects of BRAF Mutations

### 3.1. BRAF, Melanogenesis, and Phenotype Switching

Oncogenic *BRAF* controls many aspects of melanoma cell biology and the common presence of *BRAF* mutations in nevi, primary, and metastatic melanoma suggests that this phenomenon is an early event in melanomagenesis [37], although the role of BRAF as a prognostic marker is under debate [38,39]. It has been suggested that BRAF V600E-mutated melanomas show an aggressive phenotype [40], also depending on their subcellular localization [41]. Furthermore, BRAF V600K positive melanoma is considered to be more aggressive than V600E ones [42], underlying that the mutational load of BRAF-mutated melanoma should be considered as a key aspect in melanoma management [43].

**Figure 2:**
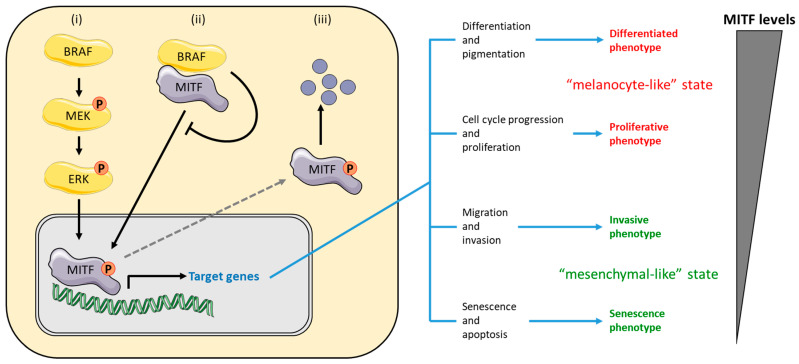
BRAF-mediated regulation of MITF, in which expression levels are linked with melanoma phenotype (see [47,50,52]), in a complex way. BRAF modulates MITF activation through several mechanisms (**left panel**): (**i**) BRAF modulates MITF activity through ERK-dependent phosphorylation; (**ii**) direct interaction between BRAF (and other RAF kinases) and MITF leads to decreased nuclear translocation, increasing cytoplasmic levels of MITF; (**iii**) ERK phosphorylation also regulates MITF degradation through the ubiquitin pathway. MITF is a driver of phenotype switching (**right panel**). Parts of the figure are drawn using pictures from Servier Medical Art (https://smart.servier.com (accessed on 17 June 2023)).

The presence of *BRAF* V600E mutation in nevus melanocytes, which have not evolved into melanomas, is associated with cell cycle arrest due to a protective mechanism known as oncogene-induced senescence [44]. Therefore, the functional relationship between BRAF, nevi, and melanoma needs to be clearly defined [45]. It has been suggested that microphthalmia-associated transcription factor (MITF), a key regulator of melanocyte differentiation, survival, and proliferation, may be responsible for the melanogenic effects of mutant BRAF [46]. Under physiological conditions, exposure to UV radiation induces the release of α-MSH from keratinocytes that binds the MC1R on melanocytes membrane activating MITF through the cAMP-PKA-CREB axis. MITF, in turn, increases the expression of proliferation and survival signals (genes such as *CDK2* and *BCL-2*) [47], melanin-related genes including *TYR*, *TYRP1*, *TYRP2*, and genes related to melanosome maturation as well as *PMEL17* and *MLANA* (MART-1 or MELAN-A) [48,49]. MITF expression is also correlated to the invasive capacity of melanoma cells and regulates the transition between proliferative and invasive states (“phenotype switching”) [50]. In fact, it is well known that low levels of MITF are linked to senescence and invasion, while high levels of MITF are linked to the proliferation and differentiation abilities of melanoma cells [51]. In this scenario, MITF can be modulated by MAPKs through (i) ERK-dependent phosphorylation, (ii) direct interaction with BRAF [52], or (iii) by controlling its degradation [47]. Consistent with these observations, treatment with CH6868398 (a MITF suppressor) potentiates the anti-proliferative effects of PLX4720 (a specific BRAFi) on melanoma cell lines [53]. The interplay between BRAF, MITF, and melanoma phenotype switching is summarized in Figure 2.

Since TYR, TYRP1, TYRP2, PMEL, and MELAN-A are recognised as melanocyte differentiation antigens (MDAs), inhibition of BRAF V600E increases MDAs expression, through the upregulation of MITF, enhancing melanoma immunogenicity [49]. In addition to the mechanisms previously described, there is further evidence of a link between BRAF mutational status and melanoma immunogenicity. Mutant BRAF, like other somatic mutations, leads to the formation of neoantigens presented by major histocompatibility complex (MHC) molecules, which may be recognised by T lymphocytes [54]. Nevertheless, BRAF V600E mediates immune escape in melanoma through several mechanisms: (i) release of immunosuppressive mediators in the tumour microenvironment (TME), such as IL-6; (ii) alteration of the composition and phenotype of neighbouring cells; (iii) downregulation of melanoma differentiation antigens expression; and iv) downregulation of MHC molecules [50,54,55]. Specifically, Bradley and colleagues reported that oncogenic BRAF activation causes an altered surface expression of MHC-I molecules (and subsequent reduction in CD8+ T-cell recognition of melanoma cells) due to their rapid internalization and subsequent sequestration within endolysosomes [56]. This event, which is not accompanied by transcriptional activation, is reverted by MAPK pathway inhibitors (MAPKi) [56].

### 3.2. BRAF Mutations and Inflammation

**Figure 3:**
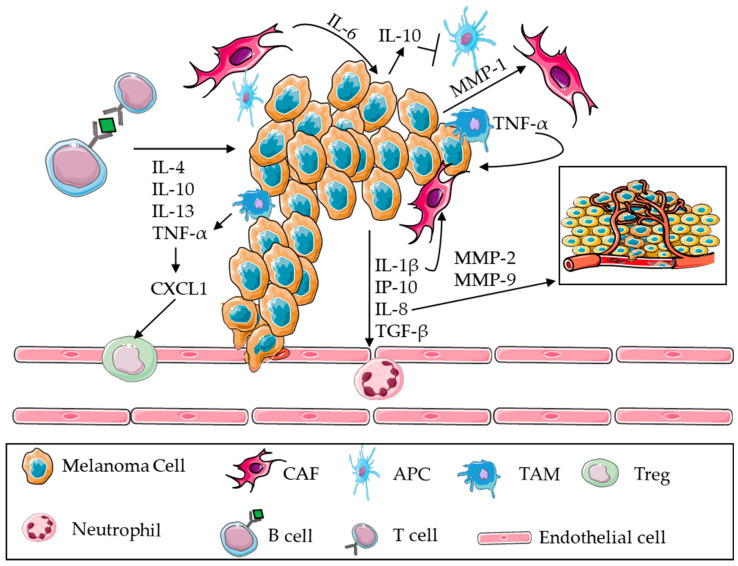
BRAF mutation and inflammation. Dynamic interaction between BRAF V600E melanoma cells and tumour microenvironment (TME) in which activated stromal cells support cancer progression. The chronic activation of the immune system leads to an accumulation of T and B lymphocytes (T and B cells). MMPs and several cytokines alter cancer-associated fibroblast (CAF) behavior; inflammation mediators like IL-1β, IL-6, IL-8, and TGF-β are responsible for neutrophil recruitment. MMP-1, produced by melanoma cells, affects CAFs and promotes interstitial collagen degradation activating tumour invasion and angiogenesis. Melanoma cells secrete IL-8-inducing MMP-2 and MMP-9 that contribute to angiogenesis and cancer vascularization. Tumour-associated macrophages (TAMs) produce TNF-α modulating CXCL1 overexpression, which enhanced recruitment of regulatory T cells (Tregs). Tregs are also recruited by TGF-β secretion. IL-6, produced by stromal cells, can promote IL-10 secretion in melanoma cells blocking the function of antigen-presenting cells (APCs). TME cells also produce IP-10 which polarized the action of immune cells. Parts of the figure are drawn using pictures from Servier Medical Art (https://smart.servier.com (accessed on 15 June 2023)).

Inflammation is an intricate set of immune, non-immune cells and mediators acting together to repair the damaged tissue and/or to respond to external injury. Chronic activation of the immune system leads to an accumulation of T- and B-cells that release pro-tumourigenic molecules (e.g., IL-4, IL-10, IL-13, and TNF-α) aiding tumour formation [57]. In particular, a dynamic interaction between cancer cells and TME, an abundant combination of fibroblasts, leukocytes, pericytes, endothelial cells, extracellular matrix, and extracellular components, influences tumour growth [58,59]. It is already known that activated stromal cells (cancer-associated fibroblasts, CAFs) support tumour progression [60]. Metalloproteinases (MMPs) and several cytokines/chemokines secreted by BRAF-mutated melanoma cells influence TME and alter the behavior of CAFs [61], that collaborate to support an immunosuppressive environment. BRAF V600E melanoma cells have been found to produce key cytokines involved in inflammation such as IL-1β, IL-6, IL-8, and TGF-β as signals, for instance, for neutrophil recruitment [62]. Whipple and Brinckerhoff demonstrated that BRAF V600E melanoma cells secrete factors that interact with stromal fibroblasts and enhance tumourigenicity [63]. The gene expression analysis in WT and BRAF V600E mutated cells revealed the induction of IL-1β, IL-6, IL-8, and MMP-1. A multiplex analysis on conditioned media from cancer cells confirmed that cytokines were upregulated by BRAF V600E and that treatment with vemurafenib (a specific BRAFi) reverted this increase. The strict interaction between cancer cells and TME alters and damages the stromal structure leading to the release of signal proteins such as SDF-1 (CXCL12) and its receptor called CXCR4; the overexpression of these molecules is associated with cancer progression [64,65]. In particular, the authors showed that an increase in IL-1β level impaired the stromal cell-mediated immunosuppression, which was reverted after vemurafenib treatment inferring that BRAF V600E drives CAF immunosuppression through IL-1β induction. Their data emphasize the protein’s role as an upstream regulator in melanoma and stromal cells, as cancer IL-1β promotes gene expression in CAFs through the secretion of growth factors and chemokines. Further evidence demonstrates that IL-1β does not seem to be controlled via an autocrine feedback loop, as the use of exogenous IL-1β does not recover the cytokine expression in melanoma cells [63]. In fact, a link has been identified between increased BRAF, MMP-1, and invasive behavior. MMP-1 promoted interstitial collagens degradation and cleaved PAR-1 by activating signal transduction and thus leading to tumour invasion and angiogenesis. These data suggest that the same occurs in adjacent fibroblasts affecting the mechanism of tumourigenicity of BRAF V600E melanoma cells. It is known that melanoma cells in the TME are the main producers of IL-8, while fibroblasts in monocultures predominantly secrete IL-6 and, when CAFs were co-cultured, IL-6 and IL-8 secretion was detectable in the media. Notably, the use of molecules that simultaneously block IL-6 and IL-8 is sufficient to fully inhibit CAF-induced human melanoma cell invasiveness [66]. Interestingly, melanoma cells and CAFs interact in a bidirectional manner and this interdependence may regulate cell growth and metastatic progression [67]. Mohapatra and colleagues investigated the mechanism according to which IL-6 was related to increased invasiveness in BRAF V600E melanoma cells [68]. They investigated the signalling of IL-6 and WNT5A, a member of the WNT family, separately or in combination, by blocking them with an antibody and an antagonist, respectively, concluding that IL-6 acts independently of WNT5A [68]. Actin reorganisation is a crucial step in cancer cell migration and invasion which are involved in the activity of the small GTPase Cdc42 in melanoma cells. Blocking IL-6 and WNT5A also blocks GTPase activity and thus actin function. Therefore, Mohapatra’s work demonstrated that the acquired resistance to BRAFi triggers a relevant increase in IL-6 secretion in BRAF V600E melanoma cells and that the inhibition of both proteins alters cell invasion capability representing a possible therapeutic target [68]. An increase in IL-8 associated with BRAF mutation has been demonstrated to be essential for the induction of MMP-2 and MMP-9 that contribute to cell migration and to angiogenesis enhancing cancer vascularization to support the oxygen retrieval and nutrient supply to cancer cells [58,69]. Strictly crosstalk between tumour-associated immune cells and cancer cells is via the cancer progression and invasion. In particular, TNF-α, produced by tumour-associated macrophages (TAMs), modulates the overexpression of CXCL1 involved in cancer development and malignant progression [70]; CXCL1 overexpression is associated with an aggressive form of melanoma [71], as it promotes tumour growth and metastasis. CXCL1 expression enhances the recruitment of Tregs to facilitate the immune escape of the tumour [72]. Tregs can indeed be enhanced by TGF-β which plays a specific role in resistance to PD-1 inhibitors [73]. As previously elucidated, IL-6 can also be produced by stromal and melanoma cells as well, but IL-6 secreted by TME cells can promote the IL-10 secretion in melanoma cells reducing the immune response and the production of pro-inflammatory cytokines and block the function of antigen-presenting cells (APCs) [74,75]. Montoyo-Pujol and colleagues analysed a large number of soluble factors in both supernatant and sera from patients: interestingly, they detected the same molecules except for CXCL1 (present only in the supernatant). This suggests a local production and PDGF-BB (only present in sera) acting as a chemotactic molecule and showing a specific role in lymphangiogenesis promoting tumour progression and metastasis [76]. Data obtained by the authors indicated that PDGF-BB is not produced by melanoma cells, suggesting a possible secretion as systemic feedback to melanoma development. The authors also explained the diagnostic and prognostic role of IP-10 (CXCL10) as a possible biomarker to distinguish the different stages of the disease, with high IP-10 levels correlating with a poor prognosis. Different cell types such as monocytes, activated neutrophils, stromal cells, and many others secrete IP-10 under IFN-γ stimulation [77]. Bagheri demonstrated the peculiar role of the CXCL10/CXCR3 axis, which is able to polarize Th1 cells and induce activation of CD8+ T cells, NK cells, and NKT cells through IFN-γ secretion [77]. Figure 3 summarizes the alteration in TME induced by BRAF-mutated melanoma.

## 4. Targeted Therapy for BRAF-Mutated Melanoma

Over the decade, clinical research has tested numerous therapeutic strategies to achieve greater efficacy and fewer side effects in late-stage melanoma. The recent use of immune checkpoint inhibitors in the therapy of advanced melanoma leads to a significant improvement in prognosis, particularly, the use of immune checkpoint inhibitors regards anti–PD-1, such as pembrolizumab/nivolumab, and anti–CTLA4, such as ipilimumab [78]. However, a high level of toxicity was found in about 60% of patients, which is linked to aberrant activation of the immune system and leads to damage to healthy tissues. At the same time, important results have also been obtained for the treatment of the metastatic phase of melanoma with drugs that can inhibit specific molecular targets.

**Figure 4:**
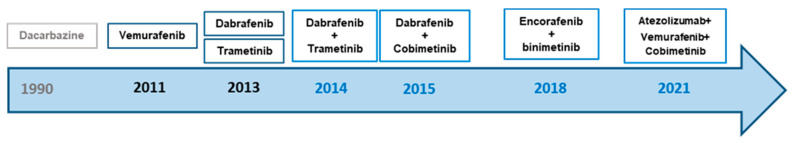
Timeline of FDA-approved targeted therapy in metastatic melanoma.

As previously stated, approximately 70% of patients with metastatic melanoma have mutations affecting oncogenes, which regulate key pathways involved in tumour progression and thus the relatively malignant phenotype. For this reason, targeted therapy represents, currently, a promising therapy for metastatic melanoma harboring drug-sensitive mutations. The target-specific therapy involves the use of small molecules and/or antibodies that specifically inhibit the mutated proteins [79] (see Figure 4).

The use of selective BRAFi includes vemurafenib (PLX4032), dabrafenib, and encorafenib. These drugs (the second generation of BRAFi) are specific kinase inhibitors that specifically bind the ATP-binding site of BRAF-mutated protein, leading to their inactivation. The biochemical affinity of BRAFi for mutated BRAF translates to potent inhibition of ERK phosphorylation and of cell proliferation exclusively in BRAF-mutant cell lines with an increase in efficacy and specificity. The Food and Drug Administration Agency (FDA) in the USA approved Vemurafenib in August 2011 for the treatment of non-resectable metastasized melanoma (https://www.cancer.gov/about-cancer/treatment/drugs/vemurafenib (accessed on 6 August 2023)). It was developed as a low-molecular-weight molecule for the inhibition of the mutated serine–threonine kinase BRAF, and it selectively binds to the ATP-binding site of BRAF V600E kinase and inhibits its activity and hence the MAPK pathway, triggering cellular apoptosis [80]. In 2011, the efficacy of vemurafenib as a monotherapy compared to dacarbazine chemotherapy showed a relative reduction of 63% in the risk of death and of 74% in the risk of tumour progression [81] and the median OS was 13.6 months compared to 9.7 months using dacarbazine [82]. Clinical trials using vemurafenib showed impressive results but, despite the initial therapeutic response observed in most patients, the early resistant relapse remains the main limit of this therapeutic strategy (as described in paragraph 5).

The resistant phenotype can also be acquired following a period of treatment with another BRAFi, dabrafenib, which was approved by the FDA in 2013. Dabrafenib is almost 20 times more specific for *BRAF* V600E mutants with respect to BRAF WT in multiple cancer cell lines with a lower IC50 in comparison with other BRAFi and displays inhibition in cell lines containing alternative oncogenic BRAF mutations [83]. Encorafenib, which acts as an ATP-competitive RAF serine/threonine kinase inhibitor, is one of the last BRAFi approved by the FDA for the treatment of metastatic melanoma. This drug has shown improved efficacy in the treatment of metastatic melanoma compared with vemurafenib [84].

The use of BRAFi monotherapy in metastatic melanoma showed a high rate of objective response and improved OS, in comparison with chemotherapy in both an in vivo xenograft mouse model and in clinical trials. However, clinical use is limited due to the acquired resistance in most of the treated patients within about 7 months after the end of the therapy [85,86,87]. Therefore, reactivation of the MAPK pathway occurs, leading to uncontrolled proliferation, including upstream activating mutations or downstream MAPK pathway alterations. For this reason, one of the new frontiers in late-stage melanoma therapy is the combination of multiple compounds capable of containing or avoiding the acquired resistance process. Drug combination therapies often aim to inhibit different transduction pathways crucial for cancer cell survival. The combined use of BRAFi and MEK inhibitors (MEKi) can inhibit the downstream MEK activation, overcoming drug resistance against BRAFi.

Trametinib is a highly selective and reversible allosteric inhibitor that regulates the activation and activity of MEK1 and MEK2 kinases [84]. In patients with advanced melanomas with *BRAF* V600 mutation, three different combinations of BRAFi plus MEKi have been shown to yield superior clinical outcomes over BRAFi alone: dabrafenib plus trametinib versus dabrafenib; vemurafenib plus cobimetinib versus vemurafenib; encorafenib plus binimetinib versus encorafenib [88,89].

**Figure 5:**
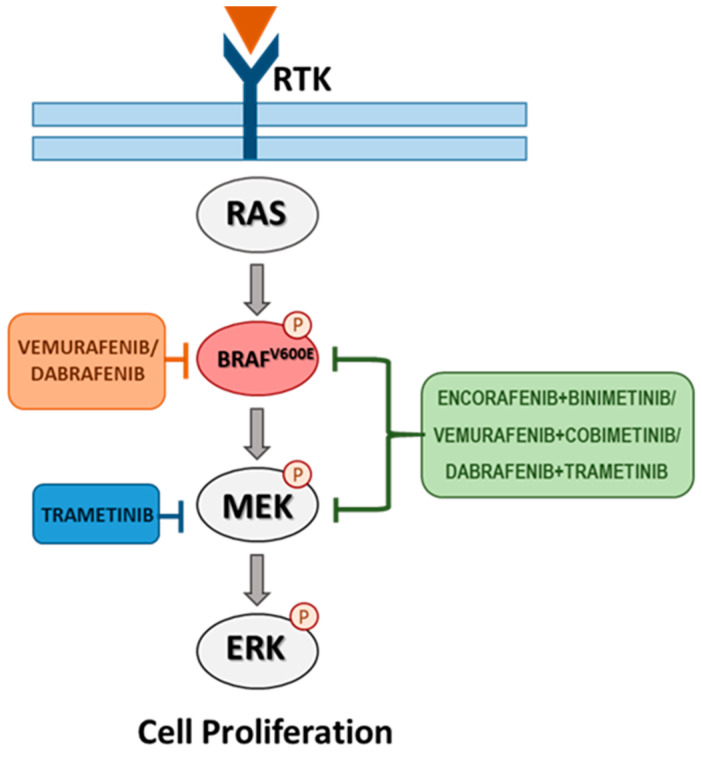
Targeted therapy (single and combined) for the treatment of metastatic melanoma. Binding of ligand with RTKs activates MAPK signalling cascade, leading to cell proliferation. In this context, vemurafenib or dabrafenib single therapy selectively inhibits BRAF V600E activity; trametinib regulates the activation of MEK kinases; BRAFi/MEKi combined therapy blocks BRAF V600E and MEK kinases.

As for the toxicity profile of vemurafenib plus cobimetinib, it differs from that of dabrafenib plus trametinib. In fact, diarrhea and nausea, are more common with vemurafenib plus cobimetinib, while pyrexia is more common with dabrafenib plus trametinib. Combined treatment with the BRAFi dabrafenib and the MEKi trametinib in patients with BRAF V600E positive melanoma showed a 76% rate of complete or partial response with combination therapy, compared to 54% observed with dabrafenib monotherapy and improved the OS and progression-free survival (PFS) of patients with unresectable metastatic melanoma compared with vemurafenib/dabrafenib monotherapy. Therefore, the results obtained demonstrate a delay in resistance development and a reduction in secondary malignancies [90] (see Figure 5).

However, despite these promising results, almost all patients (diagnosed with BRAF-mutated advanced melanoma) experience tumour relapse within several months after initiation of combined treatment. Behind this, there are different mechanisms of drug resistance, underlying the progression of the disease and the activation of both the MAPK and PI3K/AKT pathways. Indeed PI3K/AKT is the second most deregulated pathway in melanoma [91]. Drug combination therapies often aim to inhibit different transduction pathways crucial for cancer cell survival, suggesting significant functional crosstalk between the key MAPK and PI3K/AKT pathways (the inhibition of only one of these two signalling pathways often leads to compensatory activation of the other) [92]. For this reason, one of the new frontiers in late-stage melanoma therapy could be the combination of multiple compounds that can simultaneously inhibit the MAPK and PI3K/AKT pathways to overcome the acquired resistance process with synergistic effects in inducing apoptosis. Additionally, the combination of antibodies against immune targets with BRAFi/MEKi results in complementary response kinetics of these treatment approaches, suggesting that the optimal combination or sequencing of immune checkpoint inhibitors and targeted therapies may provide a way to overcome acquired resistance.

As previously mentioned, there are significant geographical variations in the prevalence of melanoma subtypes and thus in the incidences of *BRAF* mutations [29]. In fact, unlike the Caucasian population, Asian melanoma patients have a relatively low frequency (about 20%) of *BRAF* mutations [93]. Therefore, the efficacy of BRAFi/MEKi therapy or immunotherapy may differ between Western and Eastern melanoma patients [94]. These findings are of particular relevance, for example, for the management of melanoma brain metastases, since it was observed that the development of metastases was significantly higher in patients treated with BRAFi/MEKi than in those treated with immune checkpoint inhibitors [95]. Hence, the use of immunotherapy should be considered as a first-line therapy for brain metastases [96].

## 5. Resistance Mechanisms to Targeted Therapy in BRAF-Mutated Melanoma

Despite the observed improvements in clinical outcomes with therapeutic strategies based on a combination of BRAFi/MEKi, the durable benefit is limited by the development of primary or acquired resistance and subsequent disease progression [97,98,99,100]. Primary resistance mechanisms occur in approximately 50% of treatment-naïve melanoma patients [85], and are mainly associated with large intra-tumoural heterogeneity or pre-existing genetic causes: *RAC1* and *HOXD8* mutations [101]; *MEK* mutations [18]; *CCND1* amplification [102]; loss of *PTEN* [103]; *NF1* expression [104]; and mutations in the *TERT* promoter region [105]. The latter has been recently associated with MAPKi therapy response [106,107,108]. However, the mechanism underlying the potential contribution of *TERT* mutation status to MAPKi resistance needs to be clarified since conflicting results have been reported [108,109]. Alternatively, reactivation of the MAPK pathway may be due to activation of the c-jun/RHOB axis [110], increased c-MET signalling and stromal secretion of HGF [111], and suppression of BIM expression due to loss of PTEN [103]. Interestingly, since the ERK pathway regulates cell proliferation, the identification of druggable kinases involved in cell division may represent a new potential therapeutic approach [112]. Several studies have underlined how the use of kinase, cell cycle checkpoint, and mitotic inhibitors may lead to an increase in the vulnerability of melanoma cells to BRAFi/MEKi [113,114]. In most cases, however, patients develop acquired resistance after the initial higher tumour response rate due to selective pressure from targeted therapy.

Several molecular alterations responsible for the establishment of secondary resistance to BRAFi and MEKi usually lead to the reactivation of MAPK signalling, which occurs in 80% of BRAFi-resistant tumours [115]. However, activation of alternative MAPK-independent survival pathways to maintain BRAFi resistance has also been described, including secondary alterations in the PI3K/AKT/mTOR pathway [116] or presumable selection in response to treatment of pre-existing subclones that exhibit stem cell-like properties [97].

The first mechanism regulating MAPK-dependent survival signalling involves the loss of ERK-mediated feedback loops [117]. Under physiological conditions, these loops are involved in the direct modulation of signals activated by various RTKs, including EGFRs, IGFR1, PDGFR, or FGF3. Following inhibition of MAPK by BRAFi, there is a loss of negative feedback loops, leading to upregulation of PDGFRβ and EGFR receptors, which are considered critical mechanisms for adaptive resistance to BRAFi and MEKi [117,118]. Furthermore, the induction of resistance to BRAFi is associated with the downregulation of DUSP9 and SPRY2. DUSP9 causes the inactivation of ERK and activation of p38 MAPK signalling pathways [119], whereas SPRY2 is an antagonist of the FGFR-induced RAS/MAPK signal pathway [120]. Therefore, this nongenetic adaptive resistance, mediated by these negative feedback loops allows melanoma cells to evade BRAFi treatment [121,122]. These protracted feedback loops can also maintain tumour development, progression, and drug resistance by influencing the production of reactive oxygen species (ROS) and autophagy [123].

Autophagy plays a critical role in cancer cells in eliminating dysfunctional mitochondria while maintaining the normal function of mitochondria-mediated metabolism and providing needed energy through continuous glutamine metabolism. Notably, inhibition of the MAPK pathway can induce ER stress-mediated autophagy through the PERK/ATF4/TRB3 axis or IRE1/TRAF2/ASK1/JNK activation [123]. Recently, tumour progression and resistance to BRAF inhibition have also been shown to be due to autophagy promoted by the TFEB-TGF-β axis [124]. In addition, autophagy can be triggered by the upregulation of the LKB1-AMPK-ULK1 signalling axis through ERK inhibition [125].

As mentioned above, another mechanism involved in melanoma plasticity is “phenotype switching”, in which reversible transcriptional changes and epigenetic modifications induced by MITF and epithelial–mesenchymal transition transcription factors lead to a dynamic switch of cell state between two main phenotypes, the proliferative state of melanocytes and the mesenchymal invasive state [50]. The switch between distinct transcriptional programs driving the cellular plasticity of melanoma cells has been widely described as a mechanism of targeted therapy resistance [126,127]. Particularly, following BRAFi and MEKi therapy, distinct phenotypes with differential MITF activity have been described (see Figure 2), regulating the activation of transcriptional programs involved in drug resistance [128,129,130]. In detail, the “melanocyte-like” state is characterized by high expression of MITF and other transcription factors such as SOX10, ZEB2, and downstream markers (TYR, TYRP-1, and MELAN-A). The transition to the invasive “mesenchymal” state, on the other hand, is characterized by down-modulation of *MITF* (by TGF-β) and overexpression of TEADs, RTKs (*AXL*, *EGFR*), *FGF2*, *SOX9*, *ZEB1*, *POU3F2*, and *ROR2* gene, the latter being responsible for activation of the WNT5A pathway associated with a dedifferentiated state [51]. However, several studies have shown that there is an “intermediate phenotype” between the two extremes “melanocyte-like” and “mesenchymal” states, characterized by an intermediate invasive potential [131]. In this context, the interplay between tumour cells and TME appears to control phenotypic diversity and govern cell state transition. Moreover, TME modifications and autocrine/paracrine effects are strictly related to the effects of BRAFi and/or MEKi. Many works report as the targeted therapy induces senescence in cell populations not drug sensible and that this condition upregulates the expression of two different players, MMP-2 and MCP-1, with a promoting pro-metastatic mechanism by PARP-1/NF-κB activation [132,133]. Vemurafenib treatment downregulates FRA1 inducing IGF-1 and EGF that leads to the PI3K/AKT/NF-κB pathway activation. IGFBP3 binds IGF1 blocking the IGFR1 interaction, but in the TME IGFBP3 levels are decreased [134]. An interesting work underlines the role of TGF-β as an inducer of fibroblast differentiation by increasing α-SMA expression, fibronectin accumulation, and NRG1 production [98]. The role of fibroblasts in conferring tumour resistance to BRAFi through paradoxical activation of the MAPK pathway is well established [135]. Activation of the MAPK pathway is also found in cancer-associated macrophages (CAMs) via the production of VEGF [136]. Acquired BRAF resistance also affects the secretion of certain cytokines by altering their levels. In particular, it has been recently demonstrated that secretion of IL-10, VEGF, IL-1β, and IL-8, were increased in vemurafenib-resistant cells [137] as well as several chemokines as MCP-1, MIP-1α, MIP-1β, RANTES, and eotaxin, which are involved in monocytes and dendritic cell chemotaxis [137].

Interestingly, multiple examples of data from the literature suggest that BRAFi/MEKi-dependent modulation of TME leads to changes in tumour immunogenicity [138,139]. Studies of patients’ biopsy specimens showed that lymphocyte infiltrates were increased after BRAFi administration and that CD8+ were found to be reduced when tissue necrosis increased [140]. Melanoma is a highly immunogenic cancer and endogenous T cell-driven immune checkpoint control is a key player in cancer treatment. It is possible to stimulate CTLs to attack the tumour by blocking specific molecules such as the PD-1 or its ligand PD-L1 and the co-inhibitory receptors CTLA-4. An increased expression of PD-L1 is peculiar to resistant melanoma cells that are a direct consequence of the reactivation of the MAPK/PI3K pathway by c-Jun and STAT3 [141]. As previously mentioned, increased antigen-specific T-cell recognition such as MELAN-A and gp100 is closely associated with a BRAF-mutated condition [142].

Phenotypic plasticity in melanoma is also controlled by metabolic rewiring. Tumour cells exhibit altered metabolism characterized by increased reliance on aerobic glycolysis, fatty acid (FA) and nucleotide synthesis, and glutaminolysis [143]. Metabolic reprogramming is strongly influenced by the interplay of intracellular factors (mainly constitutive activation of oncogenic signalling pathways or inactivation of tumour suppressor genes) and extracellular conditions (hypoxia, acidification of the microenvironment, and nutrient deprivation) [144,145]. In the context of melanoma, the early stages of development are characterized by increased glucose uptake and glycolytic rates, as well as by significant alteration in glutamine metabolism [146,147].

Aerobic glycolysis provides a source of lactate and metabolic precursors of biomacromolecules [148], but also influences antioxidant cellular responses through the production of NADPH and glutathione via branched metabolic pathways [149]. This distinct glycolytic phenotype in BRAF-mutated melanomas is triggered by mutations in *NRAS* and *BRAF* [150]. These oncogenes are responsible for the constitutive activation of the MAPK pathway and subsequent activation of transcription factors (such as MYC and HIF-1α), as well as PI3K/AKT/mTOR and Wnt/βcatenin signalling [151,152]. These signalling pathways are in turn responsible for regulating the transcription of genes involved in glucose metabolism such as *GLUT1*, *HK2*, *LDH*, and *NAMPT* [147,153,154].

In parallel, BRAF mutations inhibit oxidative phosphorylation (OXPHOS) by suppressing the expression of MITF and its target PGC-1α [155]. However, other molecular factors are also involved in regulating the switch to the glycolytic phenotype. For example, PKM2 acts as a master regulator promoting the expression of many glycolytic enzyme genes, while the loss of PTEN, which negatively affects the PI3K/AKT pathway, contributes to the promotion of glycolytic metabolism [156]. In addition, SOX4 is involved in rewiring the glycolytic metabolism of melanoma cells by increasing the expression of the enzymes GLUT1, HK2, and LDHA [157]. More recently, Laurenzana et al. showed that the uPA/uPAR axis is involved in glycolytic reprogramming by regulating the expression of the glycolytic enzyme enolase-1 [158]. Finally, PDK, a target gene of HIF-1α, controls and inhibits the activity of PDH in the oxidation of glucose-derived pyruvate and suppresses OXPHOS [147,159]. On the other hand, adaptation to extracellular hypoxia leads to sustained activation of glycolysis and thus to environmental acidosis, which favours further growth of cells with increased glycolysis [160].

However, it is noteworthy that melanoma is a very heterogeneous tumour and subsets of melanoma exhibit a “mixed” glycolysis/OXPHOS metabolic phenotype that has the flexibility to use different energy sources (FA oxidation or glutamine) and nutrients to adapt its growth to different TME conditions [161,162]. The key molecular player in the switch of metabolism from OXPHOS to glycolysis is the BRAF/MAPK pathway, and, as described above, therapeutic strategies are currently based on a combination of their inhibition [97,163]. However, an adaptive response based on the flexibility of intracellular metabolism mitigates drug-induced stress, which limits the efficacy of targeted therapy [153,164].

In this context, melanoma cells resistant to BRAFi pressure are characterized by reduced glucose cell dependence, mitochondrial anaplerotic metabolism, and consequently increased ROS. This metabolic phenotype is dependent on the activation of PC and reactivation of the MITF/PCG-1α axis [165,166,167]. In addition, cellular redox homeostasis is also supported by amino acid biosynthetic pathways, including serine, glycine, and glutamine. The role of metabolic reprogramming towards OXPHOS at the onset of treatment resistance is confirmed in patients where lower LDH and NAMPT expression is associated with a significantly more favourable outcome [167,168]. However, a variety of metabolic mechanisms involved in drug resistance in melanoma have been described in the literature. Among others, impaired lipogenesis and cholesterol homeostasis have been reported. Indeed, an alteration in lipid composition (in particular, an increase in MUFA levels) or altered expression of lipid enzymes such as FASN, ACAT2, SOAT, SCD, SREBP-1, PGES, and others have been documented in BRAFi-resistant clones and BRAFi-resistant patients [169,170,171]. Moreover, the increased dependence of BRAFi-resistant melanoma cells on mitochondrial metabolism has been demonstrated [172,173,174].

An interesting finding reported in several studies is that FA metabolism correlates with MITF levels [175]. In addition, several studies have extensively demonstrated the driving role of the NAMPT/NAD axis or serine–glycine metabolism and glutamine metabolism (glutaminase, GLS) in the acquisition of resistance to BRAFi [176]. Collectively, these results suggest the possibility of developing a new strategy to overcome drug resistance to MAPKi in melanoma based on a combination of BRAFi/MEKi and metabolic pathway inhibitors. Preclinical data showed that molecular targeting of FASN in melanoma-resistant cell lines increased sensitivity to vemurafenib and led to increased expression of the *DHCR24* [170]. Talebi and colleagues have shown that BRAF-sensitive cells respond to BRAF inhibition by downregulating the processing of SREBP1 and thus lipogenesis, a process that is restored in resistant cells to protect them from ROS-induced damage and lipid peroxidation [176]. Recently, promising approaches for the treatment of melanoma have been proposed based on ferroptosis, a regulated cell death involving iron-dependent accumulation of lethal lipid-based ROS, particularly lipid hydroperoxides [177].

The alteration in glutamine metabolism that occurs in BRAFi-treated tumours induces histone hypermethylation, promoting BRAFi resistance [178]. Extracellular signals and TME factors influence epigenetic and transcriptional programs, such as in BRAFi-treated tumours, contributing to the development of resistance to targeted therapy, both as an adaptive response or as the emergence of drug-resistant subclones [179]. BRAFi resistance can arise from changes in the activity of histone-modifying enzymes, leading to alteration of transcriptional networks resulting in resistant phenotypes. Among the others, downregulation of the sirtuin proteins (class III HDACs) SIRT2 and SIRT6, and upregulation of SIRT1 have been reported to be associated with MAPKi resistance in melanoma [180,181,182]. Deregulation of the activity of a number of histone lysine demethylases has also been linked to targeted therapy resistance [183]. Furthermore, BRAFi resistance has been attributed to the differential expression of a set of transcriptional master regulators (e.g., FOXO, ZEB1, HIF1, STAT, and E2F) in BRAFi-resistant cells, and consequent deregulation of downstream pathways, such as MAPK, TGF-β, ADCY, and MITF signalling, in order to circumvent upstream targeted kinases [184].

A final aspect to take into consideration concerns the role of cancer stem cells (CSCs) and ncRNAs in BRAFi mechanisms of resistance. The existence of a small population of cancer cells with stem-like properties, named CSCs, resistant to standard cytotoxic therapies, such as radio- and chemotherapy, has been demonstrated [185]. Targeted therapies with BRAFi and MEKi are also unable to eliminate CSCs. These cells are able to tolerate the pharmacological inhibition of BRAF and MEK by upregulating the expression of the Hippo transducers YAP/TAZ and, consequently, enhancing ERK1/ERK2 activity [186]. Recently, SCD1 was shown to be involved in the mechanisms of drug resistance of CSCs by mediating the upregulation of YAP/TAZ [169].

**Figure 6:**
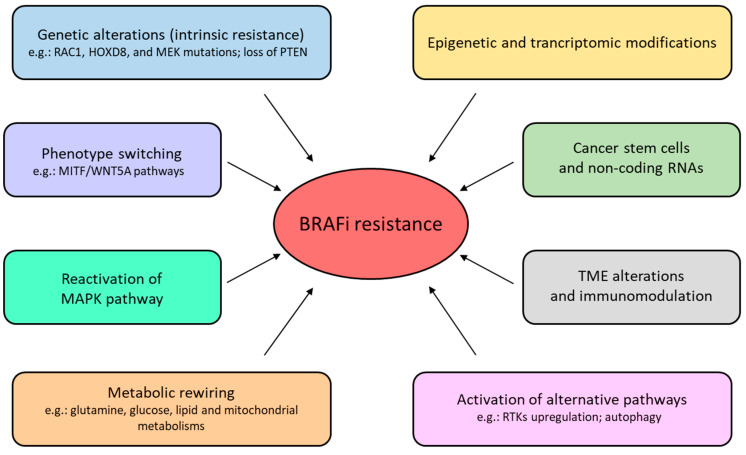
Main BRAFi resistance mechanisms addressed in this paper.

NcRNAs are involved in the development of BRAFi and MEKi resistance. The role of miRNAs is well studied and some of them promote resistance (miR-514a, miR-34a, miR-100, miR-125b, miR-1246, miR-204, miR-211, miR-4443, and miR-4488) while others (miR-7, miR-32, miR-126-3p, miR-199b 5p, miR-200c, miR-524 5p, miR-579 3p, miR-659, and miR-550a-3-5p) restore sensitivity to BRAFi and MEKi [187,188]. Altered expression of some miRNAs (e.g., miR-1246, miR-4488, and miR-4443) promotes the acquisition of resistance by regulating genes involved in autophagy, whereas others (miR-204, miR-211) do so by upregulating RAS/MEK/ERK pathway [189,190,191]. Recently, BRAFi resistance has also been attributed to several lncRNAs such as SAMMSON, MIRAT, EMICERI, and LENOX (LINC00518) [179,192]. LncRNAs can contribute to the development of resistance through a wide range of modes of action. For example, EMICERI activates a neighbouring gene MOB3B, promoting the activation of the Hippo pathway, thus conferring BRAFi resistance, whereas MIRAT regulates the MAPK pathway by binding to the MEK scaffold protein IQGAP1 [193,194]. SAMMSON interacts with p32, required for 12S ribosomal RNA processing, leading to regulation of the mitochondrial metabolism whereas LENOX interacts with the small GTPase RAP2C, promoting its interaction with DRP1 and affecting mitochondrial fission through enhanced DRP1 S637 phosphorylation [195,196]. SAMMSON, thus, operates along with LENOX by regulating OXPHOS and, subsequently, promoting MAPKi resistance [196]. The main mechanisms of BRAFi resistance described in this paragraph are summarized in Figure 6.

## 6. Circulating Biomarkers

To date, several serologic markers have been investigated as potential tools for early diagnosis, prognosis, and disease monitoring in melanoma. Actually, only LDH is a validated serum biomarker used as a strong independent prognostic factor in metastatic melanoma [197]. Recently, it has been reported that LDH and S100B represent suitable serum biomarkers in relation to disease-specific survival in metastatic melanoma. Moreover, in contrast with LDH, S100B shows a stronger correlation with response to BRAFi treatment and exhibits more accuracy in predicting progressive disease [198]. A study on candidate biomarkers proposed a preliminary pattern for enhanced risk of developing the metastasis stage in melanoma for patients that have vitamin D deficiency, doubled by an increased circulatory IL-8 and LDH [199]. Recently, Reichrath and colleagues reported that low vitamin D status is a strong predictor of poor outcomes in patients treated for advanced melanoma. However, due to the small sample size, the interpretation of the result of subgroup analysis comparing BRAF mutated and BRAF wild-type harboring tumours, makes it difficult to draw definite conclusions [200]. Therefore, despite promising inflammatory and metabolic biomarkers that have been investigated, further studies are needed to clarify their potential molecular interference with BRAF-modulated pathways.

A growing number of studies reported that tumour-derived material can be detected in body fluids, serving as “liquid biopsies”. Liquid biopsies offer a minimally invasive method for diagnosis, prognosis, and monitoring of therapeutic responses. Biomarkers such as circulating tumour cells (CTCs), circulating tumour DNA (ctDNA), and ncRNAs have been extensively studied in melanoma. Particularly, they have been applied to the BRAF gene and associated pathways, as BRAF mutation correlates with disease status and the emergence of resistance to BRAFi treatment. Hence, the identification of easily detectable biomarkers able to predict the failure of targeted therapy in advance may help clinicians in making the best therapeutic decision.

### 6.1. Non-Coding RNAs

NcRNAs may be potential biomarkers thanks to their high stability in body fluids (serum, plasma, urine, and saliva), as they are packaged in extracellular vesicles (EVs) and exosomes or complexed with RNA-binding proteins or lipoproteins, preventing their enzymatic degradation by endonucleases [187,201,202]. In particular, miRNAs and lncRNAs may represent powerful tools for predicting response or resistance to the gold standard targeted therapy, including BRAFi and/or BRAFi plus MEKi (Table 2).

However, the number of studies focusing on the identification of lncRNAs as predictors of response to targeted therapy in BRAF-mutant melanoma patients is limited. Kolenda and colleagues analyzed the association between plasma levels of lncRNAs and response to vemurafenib in BRAF-mutant metastatic melanoma patients. An association was observed between the expression of lncRNAs (Zeb2NAT, Zfas1, 7SL, and AIR) and patients’ response to vemurafenib. Patients developing progressive disease showed low expression of AIR, Zeb2NAT, and Zfas1 and high levels of 7SL [203].

On the other hand, numerous studies have identified specific miRNAs useful to monitor the responsiveness of BRAF-mutated melanoma patients to therapies. The expression of numerous miRNAs changes before and after treatment in tumour biopsies, highlighting the concept that measurement of miRNA deregulation could allow for the development of tools to predict BRAF-mutant patients’ response or resistance to BRAFi or BRAFi plus MEKi treatment [187]. In line with this, Liu and colleagues found that loss of miR-200c expression correlated with the onset of resistance to vemurafenib in clinical melanoma tissues [204]. Likewise, miR-579-3p was downregulated in tumour tissues derived from BRAF-mutated melanoma patients with acquired resistance to BRAFi and its expression correlated with a poor prognosis [205]. MiR-125a, miR-4443, and miR-4488 were upregulated, while miR-204-5p and miR-199-5p were downregulated in tumour tissues derived from BRAFi-treated melanoma patients compared to tumour tissues acquired before BRAFi treatment, supporting their potential contribution to BRAFi therapy resistance [190,206]. MiR-181a and miR-181b were also strongly downregulated in tumour tissues derived from patients before and after the development of BRAFi and MEKi resistance. In addition, the authors showed that high expression levels of these two miRNAs in melanoma tissues were associated with an increase in OS and PFS, whereas their low expression was associated with poor outcomes and marked resistance to therapy. These data suggest that these miRNAs could be potential markers to predict patients’ response to BRAFi or BRAFi plus MEKi and to monitor the onset of therapeutic resistance [207]. Several miRNAs have been detected in body fluids, such as serum and plasma, which are able to monitor the therapeutic response to BRAFi and MEKi treatment in BRAF-mutant melanoma patients. A study in which 25 metastatic BRAFV600-mutated melanoma patients treated with MAPKi were enrolled, reported that changes in plasma-derived EV miRNA levels predicted response to MAPKi therapy. Of note is that patients with high plasma levels of miR-497-5p had a better PFS compared to the patients with low expression of this miRNA. Furthermore, increased plasma levels of let-7g-5p during MAPKi treatment were associated with better disease control [208]. MiR-199b-5p and miR-4488 were identified as suitable biomarkers of melanoma patients’ resistance to BRAFi/MEKi. MiR-199b-5p and miR-4488 plasma levels were downregulated and upregulated, respectively, during disease progression compared to baseline, and displayed a good ability to discriminate pre-treatment samples from progression samples [190]. Levati and colleagues demonstrated that miR-1246 and miR-485-3p baseline plasma levels were associated with clinical response and prognosis in melanoma patients treated with BRAFi and MEKi. The miR-1246/miR-485-3p ratio is a valuable biomarker to discriminate between patients who respond to targeted therapy and who do not [209]. A recent study evaluated the expression of six circulating miRNAs in serum samples derived from 70 BRAF-mutant melanoma patients as predictors of response to therapy prior to initiation of treatments. The results showed that high levels of miR-579-3p and low levels of miR-4888 before the start of therapy were predictive factors for better PFS in melanoma patients. The relative ratio of the circulating levels of miR-4888/miR-579-3p allowed us to predict response to BRAFi/MEKi treatment in BRAF-mutant melanoma patients. Patients characterized by higher levels of this parameter had the worst therapeutic response to BRAFi and MEKi [210].

### 6.2. Circulating Tumour DNA and Circulating Tumour Cells

ctDNA represents the tumour-derived fraction of circulating free DNA in the blood resulting from necrosis and/or apoptosis. In recent years, the role of ctDNA has gained more and more attention as a promising circulating biomarker in melanoma [211]. In particular, several studies have focused on the utility of ctDNA as a predictor of treatment response, disease progression monitoring, therapy resistance, and prognosis.

Several studies demonstrated the clinical significance of plasma concentrations of ctDNA, measured in melanoma patients at baseline and/or during BRAFi/MEKi treatment [212,213,214,215,216,217,218,219]. In patients with advanced melanoma, lower basal levels of BRAF V600E ctDNA were significantly associated with longer OS and PFS than higher basal levels [214]. With regard to the predictive value of ctDNA for response to targeted therapy, different clinical studies reported that high basal ctDNA levels correlated with lower overall response rate and lower PFS to BRAFi and MEKi therapy [212,215]. Furthermore, in terms of monitoring of treatment response, BRAF V600E ctDNA concentrations decreased significantly during treatment with MAPKi in line with the response to therapy, while there was a significant increase in BRAF V600E ctDNA at disease progression [213,214]. These findings were confirmed in a large clinical validation study by Syeda and colleagues, in which a ctDNA threshold level was defined to distinguish favourable from unfavourable disease courses [219]. In this context, it became interesting to test if ctDNA could be useful to guide clinical decisions about treatment modification and optimization over time until acquired resistance develops. The Circulating Tumor DNA Guided Switch (CAcTUS; NCT03808441) phase II clinical trial aims to determine whether changes in ctDNA levels can be used to accurately inform when to switch from targeted to immune therapy in patients with BRAF-mutant melanoma, assessing the response to BRAF treatment and evaluating whether this might improve the efficacy of immune therapy [220].

In the last years, EVs have been considered a potential source of circulating DNA to improve the detection of circulating BRAF V600E in melanoma patients [221,222]. In particular, its role as a promising biomarker has been reported by Zocco and colleagues in patients with naturally occurring or therapeutically induced low levels of mutation [222]. Furthermore, BRAF V600E mutation in EV-associated nucleic acids isolated from exudative seroma of stage III melanoma patients could serve as a minimal residual disease/prognostic indicator to identify patients at risk of relapse [221].

The analysis of ctDNA as a circulating biomarker might be useful to identify the somatic mutations associated with mechanisms of resistance to targeted therapy and the identification of resistant tumour clones. As a potential mechanism of acquired resistance, it has been reported that mutation-specific ctDNA assays allow early detection of the onset of NRAS mutation in patients treated with BRAFi, other than mutations in *MAP2K1*, *AKT1*, and *PIK3CA* in melanoma patients showing treatment resistance [223,224,225].

In this context, there are different completed and ongoing clinical trials on ctDNA as a biomarker for therapy response and disease monitoring (Table 3).

CTCs are released in the peripheral blood from a primary or metastatic tumour. Their role as biomarkers has been studied in melanoma, particularly for monitoring response to targeted therapy and identifying mechanisms of resistance. It has been reported that a low count of CTCs at baseline and a decrease in CTC number after treatment initiation have been associated with response to BRAFi/MEKi therapy in metastatic melanoma patients [226,227]. Furthermore, it has been demonstrated the existence of different CTC subpopulations in melanoma is characterized by differential response to targeted therapy, allowing the identification of resistant tumour clones associated with specific mutational profiles and/or marker expression [223,228]. However, unlike ctDNA, the application of CTCs as biomarkers of melanoma in clinical practice is limited, due to their heterogeneity and the lack of standardized methodologies for their detection [229].

Therefore, the measurement of plasma concentrations of circulating biomarkers represents a valuable tool that should be considered before initiating targeted therapy, in terms of patient stratification, and over the course of treatment.

## 7. Conclusions

BRAF mutations (frequently V600E) are present in the Caucasian population in about 50% of malignant melanomas and lead to constitutive activation of the MAPK signalling pathway, which in turn mediates several phenomena, including cell proliferation, differentiation, and secretion of signal molecules, related to melanoma occurrence and progression. In particular, this review has described the impact of BRAF mutational status on melanogenesis through a mechanism strictly related to MITF, since MITF-mediated increase in melanogenesis enzymes and melanosomal markers represent key players in melanoma progression. Constitutive activation of the MAPK cascade regulates a large number of target genes including MMPs, several cytokines/chemokines, and growth factors that modify the TME in order to increase the invasive behavior of melanoma cells and alter immune cell response. The large heterogeneity of target genes explains the use of small inhibitory molecules (BRAFi) that specifically target BRAF V600 mutations leading to tumour regression. However, drug resistance limits the clinical effects of BRAFi. In this review, we described the principal mechanisms involved in primary (intrinsic) and acquired resistance, but also the adaptive response and drug tolerance to BRAFi due to tumour heterogeneity and metabolic plasticity of melanoma. Therefore, understanding the molecular and biological processes that underlie BRAFi resistance is crucial for developing new effective therapeutic strategies. To date, although treatment-related toxicity should be considered, the use of BRAFi/MEKi and BRAFi/MEKi + immune checkpoint inhibitors combinations seems to be the better strategy to overcome BRAFi resistance. The onset of resistance and the presence of toxicity indicate the need, apart from mutations in the *BRAF* gene, for additional predictive factors that allow for better stratification of patients for appropriate therapy. Therefore, the identification of circulating biomarkers, such as CTCs, ctDNA, and ncRNAs that possess the ability to select initial or alternative therapies, either minimizing side effects and optimizing treatment efficacy for melanoma patients, represent a challenge to be addressed.

## Figures and Tables

**Table 1:** Pathogenetic missense BRAF variants detected in melanoma (NCBI reference sequence number: NM_004333.6) and their frequency.

BRAF Variant	Frequency in Melanoma	Aminoacid Change
p.V600E, c.1799 T>A	70–88%	valine to glutamate
p.V600K, c.1798_1799delGTinsAA	10–20%	valine to lysine
p.V600R, c.1798_1799delGTinsAG	<5%	valine to arginine
p.V600D, c.1799_1800delTGinsAC	<5%	valine to aspartate
p.V600E2, c.1799_1800delTCinsAA	<1%	valine to glutamate
p.V600M, c.1798G>A	<1%	valine to methionine
p.V600G, c.1799T>G	<1%	valine to glycine
p.K601E, c.1801A>G	<1%	lysine to glutamate
p.D594N, c.1780G>A	<1%	aspartate to asparagine

**Table 2:** List of ncRNAs as potential predictors of response or resistance to targeted therapy (BRAFi and/or BRAFi plus MEKi).

Non-Coding RNAs	Sample Type	References
AIR Zeb2NAT Zfas1 7SL	Plasma	[203]
miR-200c	Tumour tissue	[204]
miR-579-3p	Tumour tissue	[205]
miR-125a	Tumour tissue	[206]
miR-181a miR-181b	Tumour tissue	[207]
miR-4443 miR-4488 miR-204-5p miR-199-5p	Tumour tissue Plasma	[190]
miR-497-5p let-7g-5p	Plasma	[208]
miR-1246 miR-485-3p	Plasma	[209]
miR-579-3p miR-4888	Serum	[210]

**Table 3:** Clinical studies on circulating tumour DNA (ctDNA) in melanoma patients, as biomarker of targeted therapy response and disease progression (based on ClinicalTrials.gov (accessed on 14 June 2023)).

NCT No.	Title	Status
NCT04866680	Personalized Circulating DNA Follow-up in Melanoma (PERCIMEL)	Recruiting
NCT04720768	Encorafenib, Binimetinib, and Palbociclib in BRAFmutant Metastatic Melanoma CELEBRATE	Recruiting
NCT03808441	CAcTUS—Circulating Tumour DNA Guided Switch	Recruiting
NCT03754179	Dabrafenib/Trametinib/Hydroxychloroquine for Advanced Pretreated BRAFV600 Mutant Melanoma	Unknown
NCT03416933	Therapeutic Drug Monitoring of BRAF-mutated Advanced Melanoma	Active, not recruiting
NCT03415126	A Study of ASN007 in Patients With Advanced Solid Tumours	Completed
NCT02862743	Molecular Characterization of Advanced Stage Melanoma by Blood Sampling	Completed
NCT02858921	Neoadjuvant Dabrafenib, Trametinib, and/or Pembrolizumab in BRAF Mutant Resectable Stage III Melanoma	Active, not recruiting
NCT02700763	[18F]Dabrafenib Molecular Imaging in Melanoma Brain Metastasis	Terminated
NCT02537600	Vemurafenib and Cobimetinib Combination in BRAF Mutated Melanoma With Brain Metastasis	Completed
NCT02251314	Use of Exome Sequence Analysis and Circulating Tumour in Assessing Tumour Heterogeneity in BRAF Mutant Melanoma	Completed

## Data Availability

Not applicable.

## Abbreviations

ACAT2, Acetyl-Coenzyme A acetyltransferase 2; ADCY, adenylate cyclase; AIR, antisense of IGF2R non-protein coding RNA; AMPK, AMP-activated protein kinase; ARID2, AT-rich interaction domain 2; ASK1, apoptosis signal-regulating kinase 1; ATF4, activating transcription factor 4; AXL, AXL receptor tyrosine kinase; BCL-2, B-cell lymphoma 2; BIM, Bcl-2 interacting mediator of cell death; BRAF, v-raf murine sarcoma viral oncogene homolog B1; CAFs, cancer-associated fibroblasts; cAMP, cyclic adenosine monophosphate; Cdc42, cell division cycle 42; CDK2, cyclin-dependent kinase 2; CDKN2A, cyclin-dependent kinase inhibitor 2A; C-JUN, Jun proto-oncogene, AP-1 transcription factor subunit; C-MET, MET proto-oncogene, receptor tyrosine kinase; CREB, cAMP Response Element-binding Protein; CTLA-4; Cytotoxic T-Lymphocyte Antigen 4; CXCL, C-X-C motif chemokine ligand; CXCR, C-X-C motif chemokine receptor; DHCR24, 24-dehydrocholesterol reductase; DRP1, dynamin-related protein 1; DUSP9, dual specificity phosphatase 9; E2F, E2F transcription factor; EGF, epidermal growth factor; EGFR, epidermal growth factor receptor; EMICERI, EQTN MOB3B IFNK C9orf72 enhancer RNA I; ER, endoplasmic reticulum; ERK, extracellular regulated MAP kinase; FASN, fatty acid synthase; FDA, U.S. Food and Drug Administration; FGF, fibroblast growth factor; FGFR, fibroblast growth factor receptor; FOXO, Forkhead Box O1; FRA1, FOS-related antigen 1; GLUT1, glucose transporter 1; HDAC, histone deacetylase; HGF, hepatocyte growth factor; HIF-1 α, hypoxia-inducible factor-1α; HK2, hexokinase 2; HOXD8, homeobox D8; IDH1, isocitrate dehydrogenase (NADP(+)) 1; IFN-γ, interferon-γ; IGF-1, insulin-like growth factor 1; IGFBP3, insulin-like growth factor binding protein 3; IGFR, insulin-like growth factor 1 receptor; IL, interleukin; IP-10, interferon γ-induced protein10; IQGAP1, IQ motif containing GTPase activating protein 1; IRE1, inositol requiring enzyme 1; JNK, jun N-terminal kinase; KDM, lysine demethylase; KIT, KIT proto-oncogene receptor tyrosine kinase; LDH, lactate dehydrogenase; LKB1, liver kinase B 1; MAP2K1, mitogen-activated protein kinase kinase 1; MAPK, mitogen-activated protein kinase; MART-1 (MELAN-A) melanoma antigen recognised by T cells 1; MCP2, monocyte chemoattractant protein 2; MHC, major histocompatibility complex; microRNAs, miRNAs; MIP, macrophage inflammatory protein; MIRAT, MAPK inhibitor resistance-associated transcript; MITF, melanocyte inducing transcription factor; MMP, matrix metalloproteinase; MOB3B, MOB kinase activator 3B; mTOR, mammalian target of rapamycin; MYC, MYC proto-oncogene, BHLH transcription factor; NAMPT, nicotinamide phosphoribosyltransferase; NF1, neurofibromin 1; NF-κB, nuclear factor kappa B; ncRNAs, non-coding RNAs; NK, natural killer cells; NKT, natural killer T cells; NRAS, neuroblastoma ras viral oncogene homolog; NRG1, neuregulin 1; PAR-1, protease-activated receptor 1; PARP-1, poly(ADP-ribose) polymerase 1; PC, pyruvate carboxylase; PD-1; programmed cell death receptor 1; PDGF-BB, platelet-derived growth factor-BB; PDGFR, platelet-derived growth factor receptor; PDH, pyruvate dehydrogenase; PDK, pyruvate dehydrogenase kinase; PD-L1, programmed cell death ligand 1; PERK, protein kinase R (PKR)-like endoplasmic reticulum kinase; PGC-1α, peroxisome proliferator-activated receptor-γ coactivator 1α; PGES, prostaglandin E synthase; PI3K, phosphoinositide 3-kinase; PKA, protein kinase A; PKM2, Pyruvate Kinase M1/2; PMEL17, premelanosome protein; POU3F2, POU Class 3 Homeobox 2; PTEN, phosphatase and tensin homolog; RAC1, rac family small GTPase1; RANTES, regulated upon activation, normal T cell expressed and presumably secreted; RHOB, ras homolog family member B; ROR2, receptor tyrosine kinase-like orphan receptor 2; ROS, reactive oxygen species; RTK, receptor tyrosine kinase; S100B, S100 calcium binding protein B; SAMMSON, survival associated mitochondrial melanoma specific oncogenic non-coding RNA; SCD, Stearoyl-CoA desaturase 1; SDF-1, stromal cell-derived factor 1; SOAT, sterol O-acyltransferase; SOX, SRY-related transcription factor HMG box; SPRY2, sprouty 2; SREBP-1, sterol regulator element binding protein 1; STAT-3, signal transducer and activator of transcription 3; TAMs, tumour-associated macrophages; TAZ (WWTR1) WW domain containing transcription regulator 1; TEAD, TEA domain transcription factor; TERT, telomerase reverse transcriptase; TFEB, transcription factor EB; TGF-β, transforming growth factor-β; TME, tumour microenvironment; TNF-α, tumour necrosis factor α; TP53, tumour protein p53; TRAF2, TNF receptor associated factor 2; TRB3, tribbles pseudokinase 3; Tregs, regulatory T cells; TYRP, tyrosinase-related protein; ULK1, unc-51 like autophagy activating kinase 1; uPA, plasminogen activator, urokinase; uPAR, plasminogen activator, urokinase receptor; VEGF, vascular endothelial growth factor; WNT5A, Wnt family member 5A; YAP, Yes1 associated transcriptional regulator; ZEB, zinc finger E-box binding protein; Zeb2NAT, zinc finger AE-binding homeobox 2-natural antisense transcript; Zfas, antisense to zinc finger NFX1; α-MSH, α-melanocyte-stimulating hormone; α-SMA, α-smooth muscle actin.
